# Erysipelas—Treatment with Ointment of Nitrate of Silver

**Published:** 1844-12

**Authors:** 


*Erysipelas—treatment with ointment of Nitrate of Silver.*—Erysipelas, the ambulant and stationary, both forms of frequent occurrence and often serious, are differently treated in the hospitals and in private practice. The method of Dupuytren, however, (flying blisters,) appears to be generally adopted, and its results are, in fact, sufficiently satisfactory. M. Jobert follows a different method which he finds is perfect. It consists in surrounding the erysipelatous surface with nitrate of silver ointment, and the phlogosis promptly disappears. The ointment is prepared of three degrees of strength:—the first contains four parts of the salt, the second eight, and the third twelve to thirty parts of lard. The parts selected are anointed with a small portion of the ointment, at distances of a few centimetres, each spot being of the size of a dollar, and then covered with blotting paper. A simple eruption results, but never an eschar. The medicine is absorbed, doubtless, and acts dynamically like cantharides. The antiphlogistic action of nitrate of silver is now perfectly established, and its beneficial effects in all phlogoses can no longer be doubted.—*Annales de Thérapeutique,* from *Am. Journal.*

